# Workplace resilience of nursing professionals during the COVID-19 pandemic: a mixed methods study

**DOI:** 10.1590/0034-7167-2024-0155

**Published:** 2025-09-08

**Authors:** Juliana Dal Ongaro, Patrícia Bitencourt Toscani Greco, Emanuelli Mancio Ferreira da Luz, Juliana Petri Tavares, Daiane Dal Pai, Tânia Solange Bosi de Souza Magnago

**Affiliations:** I Universidade Federal de Santa Maria. Santa Maria, Rio Grande do Sul, Brazil; II Universidade Federal de Rio Grande. Rio Grande, Rio Grande do Sul, Brazil; III Universidade Federal do Rio Grande do Sul. Porto Alegre, Rio Grande do Sul, Brazil

**Keywords:** Resilience, Psychological, Occupational Health, Nurses, COVID-19, Pandemics, Resiliencia Psicológica, Salud Laboral, Enfermeras Practicantes, COVID-19, Pandemias

## Abstract

**Objective:**

To analyze the workplace resilience of nursing professionals who worked in hospitals in southern Brazil during the COVID-19 pandemic and the associated factors.

**Methods:**

A mixed-methods study with an explanatory sequential design, conducted in four hospitals in Rio Grande do Sul. A cross-sectional study with 845 nursing workers was followed by an exploratory descriptive study involving 17 of these workers. Descriptive and inferential statistics were used for the quantitative phase, while thematic content analysis was conducted for the qualitative phase. The data were integrated through connection.

**Results:**

The integrative analysis identified three thematic areas: Nursing workers and the effects of the COVID-19 pandemic; Resilience in different work environments; and Fear and the mental health impacts of nursing professionals during the COVID-19 pandemic.

**Conclusions:**

Low to moderate resilience is associated with the challenges of the unknown, fear of exposure, the risk of COVID-19 contamination, and the impact on mental health.

## RESUMEN

**Objetivo:**

Analizar la resiliencia en el trabajo de profesionales de enfermería que trabajaron en hospitales del sur de Brasil durante la pandemia de COVID-19 y los factores asociados.

**Métodos:**

Investigación de métodos mixtos con diseño secuencial explicativo, realizada en cuatro hospitales del estado de Rio Grande do Sul. Se utilizó un estudio transversal con 845 trabajadores de enfermería, seguido de un estudio exploratorio descriptivo con 17 de estos trabajadores. Para la fase cuantitativa, se emplearon estadísticas descriptivas e inferenciales; para la fase cualitativa, se realizó un análisis de contenido temático. Los datos fueron combinados mediante conexión.

**Resultados:**

En el análisis integrador, se elaboraron tres ejes temáticos: Trabajadores de enfermería y los reflejos de la pandemia de COVID-19; La resiliencia en los diferentes escenarios laborales; y Miedo e impactos en la salud mental de los profesionales de enfermería durante la pandemia de COVID-19.

**Conclusiones:**

La baja o moderada resiliencia está relacionada con los desafíos de lo desconocido, el miedo a la exposición, el riesgo de contagio por COVID-19 y el impacto en la salud mental.

## INTRODUCTION

The year 2020 was marked by the COVID-19 pandemic, caused by SARS-CoV-2. Nursing was one of the professions with the highest number of confirmed COVID-19 cases, followed by doctors, community health workers, and pharmacists^(1)^ A study conducted in Iran showed that most of the COVID-19 cases occurred among nursing professionals (51.3%)^(2)^. According to data provided by the Nursing Observatory, from the beginning of the pandemic until June 2023, 62,029 cases of healthcare professionals infected with COVID-19 were reported in Brazil, of which 872 professionals (nurses, technicians, and nursing assistants) died due to the virus^(3)^.

The COVID-19 pandemic had significant effects on the physical health of healthcare professionals, as evidenced by the vulnerability of their work practices, with exhausting routines, readjustments, and exposure to the risk of contamination^(4)^. Additionally, the prolonged and routine use of Personal Protective Equipment (PPE) led to pressure injuries^(5)^. Mental health repercussions were also observed, causing distress, panic, and depressive symptoms, primarily due to exposure to the virus and the daily challenges of stressful situations^(6)^.

In this context, a study indicates that healthcare professionals with no experience in public health emergencies performed worse in mental health, resilience, and social support, and consequently were more likely to exhibit symptoms such as anxiety and interpersonal difficulties^(7)^. It is also important to highlight that another challenge faced by workers during this period was compassion fatigue, which occurs as a result of professionals being frequently exposed to patient suffering, stressful working conditions, and limited use of self-care measures. However, resilience has been shown to mitigate the negative aspects of compassion fatigue, both in terms of job satisfaction and the quality of healthcare delivery^(8)^.

In this context, workplace resilience represents an opportunity to work in adverse conditions while helping maintain physical and mental health, especially in the face of unexpected situations such as the COVID-19 pandemic. Workplace resilience refers to the ability to endure or overcome adversities and adapt to these situations^(9)^.

Studies show that low resilience at work is associated with symptoms such as anxiety, depression, and exhaustion in healthcare professionals^(10)^. On the other hand, high levels of resilience foster positive feelings in the workplace, such as satisfaction, professional fulfillment, and the ability to cope with adverse situations^(10)^. Thus, workplace resilience can be a factor that promotes health and well-being in workers, while reducing risks that may negatively influence mental health.

## OBJECTIVE

To analyze the workplace resilience of nursing professionals who worked in hospitals in southern Brazil during the COVID-19 pandemic and the associated factors.

## METHODS

### Ethical Aspects

The ethical principles were respected in accordance with Official Circular No. 2/2021 on research conducted in virtual environments and Resolution 466/2012 of the National Health Council. The project was approved by the Research Ethics Committee, and the approval letter was attached to the submission. Informed consent from participants was obtained electronically.

### Study Design, Period, and Location

This is a mixed-methods study with an explanatory sequential design (QUAN ⊠ QUAL). This methodological strategy allows, through the qualitative phase, for the deepening of statistically significant data from the quantitative phase In terms of weighting, priority was given to the quantitative phase. Data integration occurred through connection^(11)^.

The study was conducted in four hospital institutions, referred to in the study as HA (hospital A...), HB, HC, and HD. These four institutions were similar in that they were public reference hospitals providing care for COVID-19 patients through the Unified Health System (SUS in Portuguese) of the State of Rio Grande do Sul, as well as being teaching institutions that follow the tripartite model of higher education (teaching, research, and extension). Additionally, in terms of the institutions’ characteristics, only one is managed by the Brazilian Company of Hospital Services (EBSERH in Portuguese). The QUAN phase was conducted from August to October 2020, and the QUAL phase from November 2022 to March 2023.

The STROBE (Strengthening the Reporting of Observational Studies in Epidemiology), COREQ (Consolidated Criteria for Reporting Qualitative Research), and MMAT (Mixed Methods Appraisal Tool) guidelines (version 2018) were followed.

### Population, Inclusion and Exclusion Criteria

The eligible population consisted of nursing professionals (nurses, technicians, and nursing assistants) distributed across 2,278 at HC, 952 at HD, 2,962 at HA, and 707 at HB. Based on sample size calculations using the Power and Sample Size for Health Researchers software, version 0.1.5^(12)^, the minimum sample size for the project was set at 534 participants. After the questionnaires were sent via email or What-sApp, the final sample consisted of 845 nursing professionals. For the qualitative phase, participants were selected through convenience sampling. Those who had provided their email addresses during the first phase of the study were invited. Theoretical saturation was reached when responses became repetitive, totaling 17 participants in this phase. In this study, theoretical saturation was understood as the point in qualitative data analysis where the researcher determines, through data analysis and the established sample, that no new facts have emerged and all relevant theories and concepts have been reached according to the study’s objectives. That is, the concepts and related aspects were identified, confirming that no additional data were needed to address the research question^(13, 14)^.

Nursing professionals working in hospital care during the COVID-19 period were selected. For the qualitative phase, participants who had provided their email addresses in the previous phase were selected. Workers who were absent for any reason during data collection were excluded.

### Study Protocol

The first phase (QUAN) was conducted via the Google Forms platform, which included sociodemographic, labor-related, and health habits information, as well as the Self-Report Questionnaire – 20 (SRQ-20)^(15)^ and the Resilience at Work Scale 25-Brazil (RAW Scale 25 – Brasil)^(16)^. For this phase, an instrument was used to collect general worker data, including sociodemographic variables (age range, gender, marital status, and number of children); labor-related variables (position, institution of employment, type of employment contract, other affiliations, managerial position, work shift, reassignment to a different department, work in COVID-19 units, and care of COVID-19 patients); and health habits (smoking, increased alcohol consumption, physical activity, medication started during the pandemic, need to take leave for health reasons during the pandemic, leave due to a COVID-19 diagnosis, leave due to suspected COVID-19, fear of exposure to the risk of contamination, physical health impact, and mental health impact).

The SRQ-20^(15)^ assesses symptoms such as depressive-anxious mood, somatic symptoms, decreased vital energy, and depressive thoughts^(15)^. It is a dichotomous scale (yes/no) consisting of 20 questions, with scores ranging from zero (no likelihood) to 20 (extreme likelihood). The RAW Scale 25 – Brasil^(16)^ assesses workplace resilience through 25 items distributed across seven dimensions: living authentically, finding one’s calling, maintaining balance, managing stress, interacting cooperatively, staying healthy, and building networks. Responses are measured using a seven-point Likert scale, ranging from zero (Strongly Disagree) to six (Strongly Agree)^(16, 17)^. Items 9 and 11 are reversed in the analysis^(17)^.

The second phase of the study (QUAL) was conducted through interviews using a semi-structured interview guide, focused on significant associations with workplace resilience. The interviews were conducted by a single interviewer, online, via the Google Meet platform, and recorded in digital format, with an average duration of 20 minutes. The interviews were then transcribed verbatim.

### Analysis of Results and Statistics

The analysis of the variables, after checking for errors and potential inconsistencies, was performed using PASW Statistics® (Predictive Analytics Software, SPSS Inc., Chicago, USA), version 18.0. Sociodemographic, labor-related, health habits, and health variables were analyzed using descriptive statistics. For the SRQ-20, the cutoff for the suspicion of Minor Psychological Disorders (MPD) was set at ≥ 7 for both genders, female and male. For the analysis of the RAW Scale 25-Brazil, the authors’ guidelines were followed^(17)^. The workplace resilience variable was categorized into “Low,” “Moderate,” and “High” levels.

For the bivariate analysis between independent variables (sociodemographic, labor-related, health habits, and MPD) and the dependent variable (workplace resilience), the Pearson Chisquare test (for categorical variables) and Spearman correlation (for quantitative variables) were used. Associations were considered statistically significant when p < 0.05.

For the multivariate analysis, Poisson regression was applied, with adjusted and robust variance, as well as the Prevalence Ratio (PR) and the corresponding confidence intervals (CI 95%). The dependent variable was dichotomized into “Low/Moderate” and “High” levels of workplace resilience. Confounding variables were included in the model when they presented a p-value ≤ 0.25 for both exposure and outcome.

In the QUAL phase, thematic content analysis was used ^(14)^. Participants were identified with the letter “E” for Nurse, “TE” for Nursing Technician, and “AE” for Nursing Assistant, followed by the numerical order of the interviews (Example: E01, E02...; TE01, TE02...; and AE01, AE02...).

The data integration was performed through connection, meaning that the interview transcripts provided depth and insights into the significant findings of the quantitative phase^(11)^. The meta-inferences resulting from the combination (QUAN ⊠ QUAL) were presented using a joint-display (Chart 1).

**Chart 1:** Joint-display of the connection between QUAN results (n=845) and QUAL results (n=17) on workplace resilience in hospital nursing professionals during the COVID-19 pandemic. Rio Grande do Sul, Brazil, 2020-2022

Thematic Axis	QUAN Results	QUAL Results	Meta-inferences (QUAN i QUAL)
	Factors associated with low workplace resilience:	Semi-structured Interview	
Theme I - Nursing Workers and the Impacts of the COVID-19 Pandemic	- Suspicion of MPD (p < 0.001)	*“This year, what I experienced was mainly a lack of energy, irritability, and chronic fatigue, you know, with no energy. Sometimes I have pains, and I feel completely exhausted, in the sense of having no patience, you know?” (E05, p. 8)* *“The truth is, I didn’t even know I had so much [resilience], I think it’s courage, strength. I didn’t even know. Because at first, I thought I wouldn’t be able to overcome all of that. But then I realized, wow, I’m capable! I felt that way, and I think it even made me stronger.” (AE01, p. 6)*	Nursing professionals with suspicion of MPD were associated with low/moderate levels of workplace resilience. This association was confirmed through the data connection, meaning that the QUAN data (SRQ20) were reaffirmed when nursing professionals explained (QUAL) that the suspicion of MPD occurred due to symptoms of fatigue, exhaustion, irritability, and a sense of ‘burnout,’ which were associated with moderate workplace resilience. The connection became evident through characteristic aspects of the domains of maintaining balance and managing stress. These were present in the feeling of being capable, having strength, and maintaining focus, even in the face of the adversities brought about by the pandemic, which may explain the moderate level of workplace resilience.
Theme II - Resilience in Different Work Environments	- Belonging to institution HA (p = 0.022)- Belonging to institution HD (p = 0.024)	-Institution HA *Well, I worked in an annex of the institution, and during the pandemic, they said they needed us in the emergency department. And that’s the department I dislike the most, I don’t like emergency. I told a supervisor, ‘Put me anywhere, but not there because it’s very disorganized.’ So everything changed completely, because it was a different routine. I didn’t feel well because it wasn’t my department, and it’s not what I usually do, but if I had to stay there, it’s not for me.* (AE01, p. 2) *[...] The nature of the work is becoming more difficult, the procedures more complex. And there’s the added challenge of working at night; I don’t have the same amount of energy. We don’t always manage to take breaks. When we lie down for a break, the phone rings, and that’s the end of the break. And it makes a difference; there’s a lot of physical effort, walking around, solving things, and there’s the stress, there’s a very critical patient, there’s pressure, there’s a team on top of you, and all the things you have to handle in that process. And in the early morning, you’re already tired. Your thinking doesn’t function well, you have to use extra energy, and that’s exhausting. (E08, p. 15)* - Institution HD *There was a time we were helping in the emergency room, and it was a stressful period because we came from another department with a very different routine. On the day I was on shift, there were five patients on mechanical ventilation, and they weren’t COVID patients, they were clinical ER patients. I remember it caused a lot of fatigue, stress, and strain on the team from that shift. (E02, p. 11) No, the institution didn’t replace anyone. Occasionally, they sent a nurse, but they didn’t have experience with the routine or with caring for oncology patients. So, I felt very overwhelmed. (E04, p. 11)*	Low/moderate levels of workplace resilience were associated with nursing professionals who belonged to institutions HA and HD. This finding aligns with the testimonies regarding the particularities that encompass the respective domains: living authentically, interacting cooperatively, and maintaining balance. Among these, it is possible to clarify the movement between different departments, the temporary ‘break’ in the bond formed in the current department, and the lack of institutional support due to the failure to replace staff during leave, which caused overload and strain on the team; issues that did not align with their personal values in relation to their work practices; and the lack of specialized professionals for such labor demands, due to the emergency hiring of new staff to meet the high demand caused by the pandemic, which at times failed to consider their experience or qualifications in a particular specialty. The challenges posed by these changes are relevant aspects for professionals to develop resilient behaviors in response to specific work situations.
Theme III - Fear and the impacts on the mental health of nursing professionals during the COVID-19 Pandemic	- Rarely feeling fear in the face of exposure to the risk of coronavirus contamination (p = 0.032) - Intense impact on mental health (p = 0.014) - High impact on mental health (p = 0.006)	*In general, fear is always present, especially because we don’t know how our bodies will react. We saw many people test positive, but they didn’t have any complications beyond flu-like symptoms, while others had to be hospitalized and either passed away or were left with long-term effects. (TE01, p. 8)* *Concerned, afraid of not being able to manage, and the mental exhaustion is immense as well, because we end up forgetting to do things, fearing that we’ll make a mistake that could have negative consequences. So, mental fatigue has been affecting us a lot. (E01, p. 7)* *Our biggest problem was psychological, the fear of making a mistake due to exhaustion. It wasn’t easy to adapt [...]. (TE07, p. 3)*	Rarely feeling fear in the face of exposure to the risk of coronavirus contamination was associated with low/moderate levels of workplace resilience. This association was confirmed in the testimonies, where professionals reported that the fear was not centered on self-contamination but rather on the transmission of the virus. This demonstrates that, when rarely experiencing the feeling of fear, nursing professionals did not invest psychic energy in developing strategies for adapting to work and daily life, including the need for personal/emotional self-care. Nursing professionals who experienced intense and high impacts on their mental health had low/moderate levels of workplace resilience. This evidence was corroborated in testimonies where ‘mental fatigue,’ excessive worry, and fear of not being able to cope led to moderate adaptation to the adverse situations that the pandemic work environment created in healthcare services. These findings also align with the ’maintaining balance’ domain, which emphasizes the need to manage the negative aspects experienced at work in order to foster resilient thinking and cope with the adversities of the moment, such as the fear of contamination by COVID-19, resulting in a significant impact on mental health.

## RESULTS

The QUAN phase showed that, of the 845 participants, nursing technicians or assistants predominated (55.6%), followed by nurses (44.4%). Of these, 18.3% were from institution HA, 10.7% from HB, 43.4% from HC, and 27.6% from HD. The majority were female (84.9%), aged between 20 and 41 years (50.2%), married or living with a partner (74%), and had children (69.0%).

Nursing professionals were classified with low (14%), moderate (55.1%), and high levels of workplace resilience (30.9%). The participants showed moderate levels of workplace resilience, as evidenced by the domains of the RAW Scale 25 – Brazil, with the following percentages: 52.1% for living authentically, 56.3% for finding their calling, 56.0% for maintaining balance, 47.7% for managing stress, 58.5% for interacting cooperatively, 44.0% for staying healthy, and 55.5% for building networks.

In the bivariate analysis, the age range of 42 to 66 years (55.2%; p = 0.023) and professionals working at institution HD (60.5%; p = 0.001) showed statistically significant differences for moderate levels of workplace resilience. Additionally, not practicing physical activity (56.9%; p < 0.000), increased alcohol consumption (55.5%; p = 0.002), not taking new medications during the pandemic (55.5%; p < 0.000), frequent fear of exposure to contamination risk (62.2%; p < 0.000), high physical health impact (60.0%; p < 0.000), high mental health impact (62.1%; p < 0.000), and poor sleep quality (58.7%; p < 0.001) were significantly associated with moderate levels of workplace resilience. The adjusted and unadjusted associations between workplace resilience, MPD, and potential confounders are presented in Table 1.

**Table 1:** Unadjusted and adjusted analyses of statistically significant associations between workplace resilience, minor psychological disorders, and potential confounders. Rio Grande do Sul, Brazil, 2020

Variables	PRb	CI (95%)	PRajl*	CI (95%)	PRaj2**	CI (95%)	PRaj3***	CI (95%)
MPD								
Yes	1,20	1,16-1,24	1,18	1,13-1,23	1,16	1,12-1,22	1,17	1,13-1,22
No	1	-	1	-	1	-	1	-
Work leave during the pandemic								
Yes	1,02	0,98-1,06	0,99	0,96-1,03	-	-	-	-
No	1	-	1	-	-	-	-	-
Work leave due to COVID-19								
Yes	1,06	0,99-1,13	1,05	0,99-1,12	1,05	0,99-1,11	-	-
No	1	-	1	-	1	-	-	-
Gender								
Female	1,05	0,99-1,11	1,06	0,99-1,12	-	-	-	-
Male	1	-	1	-	-	-	-	-
Age range								
20 to 37	1,43	0,99-1,09	1,05	0,99-1,09	-	-	-	-
38 to 44	1,02	0,98-1,07	1,03	0,97-1,08	-	-	-	-
45 to 66	1	-	1	-	-	-	-	-
Institution where they work								
HA	1,07	1,02-1,12	1,06	1,01-1,11	1,06	1,01-1,06	1,06	1,00-1,11
HB	1,04	0,98-1,11	1,04	0,97-1,10	1,03	0,97-1,09	1,03	0,98-1,09
HD	1,02	0,98-1,07	1,03	0,97-1,08	1,06	1,01-1,10	1,01	1,00-1,09
HC	1	-	1	-	1	-	1	-
Work shift								
No fixed shift	1,02	0,94-1,11	1,04	0,96-1,13	1,00	0,92-1,09	-	-
Night	0,96	0,92-1,00	0,97	0,93-1,01	0,99	0,95-1,03	-	-
Day	1	-	1	-	1	-	-	-
Fear felt in the face of exposure to contamination risk								
Frequently	1,24	1,09-1,40	1,25	1,10-1,41	1,08	0,96-1,22	1,11	0,99-1,26
Rarely	1,18	1,03-1,34	1,18	1,03-1,34	1,11	0,98-1,26	1,15	1,01-1,30
Occasionally	1,16	1,02-1,31	1,16	1,03-1,31	1,07	0,95-1,21	1,09	0,97-1,24
A lot	1,23	1,09-1,39	1,24	1,09-1,39	1,06	0,93-1,19	1,08	0,95-1,22
None	1	-	1	-	1	-	1	-
Physical activity								
No	1,08	1,04-1,13	1,06	1,02-1,10	1,03	0,99-1,07	-	-
Yes	1	-	1	-	1	-	-	-
Increase in alcohol consumption during the pandemic								
Yes	1,05	1,01-1,09	1,05	1,01-1,09	1,03	0,99-1,07	-	-
No	1	-	1	-	1	-	-	-
Medication started during the pandemic								
Yes	1,06	1,01-1,09	0,99	0,96-1,04	-	-	-	-
No	1	-	1	-	-	-	-	-
Impact on physical health								
Intense	1,17	1,07-1,30	1,14	0,94-1,15	1,04	0,95-1,15	-	-
Low	1,13	1,01-1,27	1,02	0,99-1,23	1,09	0,98-1,21	-	-
Moderate	1,15	1,04-1,27	1,08	0,98-1,19	1,08	0,98-1,19	-	-
High	1,16	1,05-1,29	1,01	0,91-1,12	1,00	0,90-1,12	-	-
None	1	-	1	-	1	-	-	-
Impact on mental health								
Intense	1,23	1,12-1,35	1,14	1,03-1,26	1,15	1,04-1,28	1.13	1,03-1,25
None	0,98	0,84-1,14	1,01	0,87-1,18	1,05	0,89-1,24	1,00	0,86-1,18
Moderate	1,11	1,00-1,22	1,09	0,99-1,19	1,09	0,99-1,21	1,08	0,98-1,19
High	1,20	1,09-1,32	1,14	1,04-1,26	1,14	1,03-1,26	1,14	1,04-1,25
Low	1	-	1	-	1	-	1	-
Sleep quality								
Poor	1,26	1,14-1,39	1,22	1,10-1,34	1,09	0,99-1,21	-	-
Bad	1,19	1,08-1,30	1,15	1,05-1,27	1,05	0,96-1,15	-	-
Fair	1,15	1,05-1,26	1,23	1,03-1,23	1,03	0,95-1,31	-	-
Good	1,11	1,01-1,22	1,09	0,99-1,19	1,05	0,95-1,15	-	-
Excellent	1	-	1	-	1	-	-	-

*Legend: MPD: Minor Psychological Disorders; PRb: Crude Prevalence Ratio. PRaj: Adjusted Prevalence Ratio. CI (95%): Confidence Interval.*

Note: PRaj1* = MPD + work leave during the pandemic + work leave due to COVID-19 + age range + institution where they work + work shift + reassigned department + physical activity + increased alcohol consumption + medication started during the pandemic + caring for COVID-19 patients + fear felt due to exposure to contamination risk + impact on physical health + impact on mental health + sleep quality; PRaj2** = Work leave + MPD + workplace resilience + institution where they work + work shift + medication started during the pandemic + impact on physical health + impact on mental health; PRaj3*** = MPD + institution where they work + medication started during the pandemic + impact on physical health + impact on mental health.

Regarding MPD, nursing professionals with suspected cases during the COVID-19 pandemic had a 17% higher prevalence of low workplace resilience. Furthermore, professionals from institutions HA and HD showed 6% and 1% higher prevalence rates for low workplace resilience, respectively, when compared to institution HC.

Nursing professionals who reported rarely feeling fear in the face of exposure to the risk of COVID-19 contamination showed a 15% higher prevalence of low workplace resilience. Professionals who reported intense and high mental health impacts showed a 13% and 14% higher prevalence of low workplace resilience, respectively.

In the qualitative phase, female participants predominated (82.3%), with an age range of 40 to 50 years (47.0%) and 5 to 7 years of work experience (64.7%). Among them, eight were nurses, eight were technicians, and one was a nursing assistant. Furthermore, three professionals were from institution HA, two from HB, six from HC, and six from HD. Additionally, eight nursing professionals reported not needing to be reassigned to a different department/unit, four were reassigned when necessary, and five were reassigned specifically to meet the demands of the COVID-19 pandemic.

The qualitative data led to the construction of two thematic categories: “The Impact of Coping with the COVID-19 Pandemic on the Health of Nursing Professionals” and “Ability to Adapt and Strategies for Maintaining Health During the COVID-19 Pandemic.” The first category highlighted the impacts of a new routine, including the need for reassignment to new work sectors with insufficient human resources, excessive working hours, and a shortage of PPE, particularly at the beginning of the pandemic. It also revealed the repercussions of stress, driven by fear and exhaustion due to concerns about transmitting the virus to family members.

*Too much overload for everyone. It was uncontrollable because the shift was supposed to have 20 employees, but when we showed up for work, there were five fewer. There was no way to replace those five at that moment. So, we worked with what we had. The institution began to close units because there was not enough workforce to help, and the replacements were not workers with the necessary knowledge, just help.* (TE08; p.4)

*I feel fatigued, demotivated [...] it’s not always to the point of depression, I love my job, I like to go, but sometimes I feel tired.* (E02; p.10)

In the second category, two subcategories were developed. The first, “The Role of Resilience in Maintaining Occupational and Personal Well-being,” highlighted aspects of the importance of resilience for maintaining both workplace and personal well-being, as reflected in the following statements:

*I think that we, one another, strengthen ourselves because we have to take care of each other, and we have to be well to move forward. So, I think we strengthen each other even more as a team, supporting one another, and those who had COVID, we would worry, call, and try to follow up, even from a distance.* (E03; p.5)

[...] *there are moments when you feel emotionally shaken, but we have incredible resilience. There is a greater strength that allows you to fight and face things again. We are reborn every day. I think it’s really resilience. It’s about finding strength, about being strong in difficult moments.* (TE08; p.12)

The second subcategory, “Strategies for Promoting Health in the Context of the Pandemic,” pointed out that practices such as reading, seeking to study new content, crafting, meditation, and engaging in physical activity at home were strategies used to “keep the body active.” These activities helped maintain focus, concentration, and a healthy routine from both a physical and mental standpoint.

*I started practicing meditation and noticed it helped me focus more on work, so that the moment we were going through wouldn’t interfere with my health* [...] *So, I was able to work more with my body and use my mind together.* (AE01; p.4)

[...] *I would read and do things: physical exercise, relaxation at home, but prayer to maintain my faith was a strong point for me.* (TE07; p.7)

In Chart 1, the joint-display is presented with the combination of results through connection (QUAN B QUAL). From this, meta-inferences and unique insights were obtained regarding workplace resilience and the significant associations between exposure factors in hospital nursing professionals during the COVID-19 pandemic.

## DISCUSSION

The data integration was consistent with the suspicion of MPD, in which nursing professionals exhibited low/moderate levels of workplace resilience (p < 0.001). The participants reported fatigue, irritability, lack of concentration and energy, and physical and psychological exhaustion. These are signs and symptoms consistent with the suspicion of MPD^(15)^.

MPD symptoms were also present concerning exposure and the risk of contamination, as well as the vulnerability to serious illness among the nursing professionals investigated. Brazilian studies with the same population and context also revealed concerning signs and symptoms of MPD, with prevalences ranging from 35.5%^(18)^ to 60.6%^(19)^. It is important to consider that the pandemic context likely contributed to the worsening of work-related illnesses among nursing professionals, as even before the pandemic, there was already a negative health scenario for these professionals, with consequences for both physical and mental health.

An Iranian study showed that professionals lacking resilience in the workplace are six times more likely to develop some form of psychological disorder (p = 0.02)^(20)^. Anxiety^(21)^, emotional burnout, and depersonalization^(22)^ are prominent examples. Developing the ability to adapt to adverse situations, such as those experienced during the COVID-19 pandemic, allows nursing professionals to minimize negative effects, improving sleep quality and increasing job satisfaction^(23, 24)^.

Moreover, resilience is a crucial factor in maintaining adaptability when faced with new challenges and adversities, which can have both personal and professional implications. This adaptive process requires stress management, recognizing personal strengths, and applying them in the workplace, as well as building support networks, whether in the workplace or in personal life, and seeking moments that favor physical and mental health. Thus, resilience assessment becomes an important strategy for workers’ self-awareness, enabling institutions to plan actions that promote resilience, health, and quality of life^(16)^.

In the integrative phase, it was initially noted that aspects of the domains “Maintaining Balance” and “Managing Stress” corroborated the low/moderate levels of workplace resilience observed in the professionals. These domains reflect characteristics such as maintaining focus, managing adverse situations, maintaining routines, and balancing professional and personal life^(25)^, even in the face of challenges, such as during the pandemic. The completely unknown scenario at that time required nursing professionals to adapt to new work processes, intensify care activities, and manage high psychological demands, in which physical and mental fatigue became evident. This may be linked to the level of resilience exhibited by these professionals.

Regarding the evidence that low/moderate levels of workplace resilience were associated with professionals from institutions HA and HD, it is important to note that both institutions specialize in providing 100% care through the Unified Health System (SUS). In this context, various changes were inevitable for these public health institutions to face the sanitary and economic crisis they encountered.

The findings from the qualitative phase supported the quantitative results and also provided contextual insights related to the work environment. Specifically, aspects such as the ability to recognize and maintain personal values and strengths, a good level of emotional awareness and regulation in the workplace, obtaining mutual support among colleagues, and the ability to overcome setbacks, improve skills, stay updated with specialized knowledge, and expand networks align with the domains of living authentically, maintaining balance, and interacting cooperatively^(25)^.

It became evident that professionals faced difficulties when reassigned to different work sectors, particularly when there was no identification with the new sector or when there was a lack of specific knowledge for the new role. This led to insecurity, overload, and burnout. Additionally, the reassignment of professionals in high-risk groups resulted in emergency hiring and the dissolution of teams, which, in turn, required the establishment of new bonds and support networks, impacting the development of resilient behaviors.

A study highlights that the disruption of existing emotional bonds within work teams, due to new organizational restructuring and professional redistribution, created a sensitive moment with implications for the social support that had previously existed. This required the formation of new bonds and the reorganization of work with new colleagues^(26)^.

Moreover, work experience (p = 0.01) and area of activity showed a significant and positive relationship with workplace resilience in nursing^(27)^. Professional unpreparedness in the face of adverse situations contributes to the emergence of stressful conditions, preventing professionals from progressing and refining their skills, which could facilitate more resilient adaptation. Furthermore, replacing colleagues to address demands outside their routine can increase the risk of contamination and transmission, representing a stressor among workers^(28)^, thereby compromising the quality of care provided to the population^(29)^. It is important to clarify that workplace resilience can enable professionals to provide safer care in the face of an unknown scenario, such as the one experienced during the COVID-19 pandemic^(30)^.

Additionally, the results revealed that nursing professionals who reported rarely feeling fear of exposure to the risk of coronavirus contamination (p = 0.032), as well as those reporting intense (p = 0.014) and high (p = 0.006) mental health impacts, showed higher prevalences of low/moderate workplace resilience. These data are corroborated by the participants’ statements, where the fear was not centered on daily exposure but rather on moments of uncertainty regarding patient care, not knowing patients’ prior diagnoses, which raised concerns about transmitting the virus to family members. This finding suggests that the feeling of fear during the COVID-19 pandemic may not have provided nursing professionals with a protective impulse, given the adaptation strategies aimed at work. Mental fatigue was also observed as an obstacle to adapting to adverse situations in the work environment during the pandemic.

In line with the evidence presented, a study showed that the fear of transmitting the disease to family members or friends, perceived uncertainty about treatment, and the unavailability of resources such as rapid tests and PPE were triggering factors for distress among nursing professionals, who were predominantly affected^(31)^. Additionally, a multicenter study involving nurses working in reference hospitals for COVID-19 care identified that fear of exposure to contamination risk was associated with the development of MPD (p < 0.001)^(19)^. In other words, factors such as fear became potential enhancers of negative impacts on the mental health of these professionals^(32)^, preventing them from making positive adaptations in their work lives.

Furthermore, mental health repercussions were evident with the emergence of burnout syndrome (p = 0.007), severe anxiety symptoms (PR = 1.36; p = 0.019), and depression (PR = 1.40, p = 0.011) in nursing professionals working directly on the front lines of the pandemic^(33)^.

The findings of this study align with characteristics related to the “maintaining balance” domain of the RAW Scale. This domain describes the need for professionals to manage the negativity experienced at work and, thereby, enhance their resilience to cope with the adversities of the moment^(25)^. In other words, the fear of exposure to contamination, experienced during the COVID-19 pandemic, resulted in a mental health impact on nursing professionals, who, in some cases, were unable to develop adaptations—either personal or professional—that could help them cope with the challenges of the moment.

A study with nursing professionals providing direct care to patients during COVID-19 across all regions of Brazil showed that low resilience levels were linked to higher average depression scores^(34)^. In summary, it demonstrates resilience as an essential factor for protecting occupational health, reducing impacts such as emotional exhaustion, physical fatigue, overload, fears, concerns, and uncertainties arising from a context like that of COVID-19. In line with this, healthcare professionals with high levels of workplace resilience were able to adapt to the adverse situations imposed by the vast COVID-19 pandemic^(10)^.

A Chinese study evaluated methods to mitigate the psychological impacts of working during the COVID-19 pandemic within the nursing team. These included measures for early assessments and interventions to alleviate anxiety, stress, and tension; promoting the creation of bonds and developing harmonious work relationships to prevent stress; and improving the social support received^(35)^. In this perspective, the importance of promoting positive aspects in the workplace emerges. Among these, resilience is highlighted, considering the negative psychological repercussions arising from the work environment, especially during the pandemic. These repercussions can lead to harm, both in individual quality of life and in the care provided to individuals, families, and the community.

### Study Limitations

A limitation of this study is the time gap between the quantitative and qualitative phases. The interviews were conducted entirely online; however, the demand for remote interactions began to overwhelm the professionals, who were often unwilling to participate or remain in front of a computer or other digital devices. Therefore, self-reporting may have been affected by the virtual nature of the study. Nevertheless, by utilizing both quantitative and qualitative methods, the inherent weaknesses of each were minimized, as the strengths of one approach compensated for the limitations of the other^(11)^.

### Contributions to the Field of Nursing, Health, or Public Policy

The evidence from this research offers important contributions to the fields of nursing and health, considering the situational diagnosis of an unexpected work reality for both nursing professionals and society. Additionally, the study highlights workplace resilience as an essential factor for overcoming adversities in nursing practice.

It is important to reflect on strategies that can contribute to the field of health and nursing, enabling partnerships between healthcare services and the formulation of public policies, with an emphasis on the comprehensive care of these professionals. In work environments, strategies for promoting resilience and well-being based on evidence should be considered, such as the use of mindfulness and cognitive-behavioral therapy^(36)^, which have been shown to be important for increasing resilience levels^(37)^, ultimately benefiting workers’ health. Moreover, it is essential for institutional management to seek measures that promote healthy work environments and foster more positive workplace performance.

## CONCLUSIONS

Nursing professionals working in the hospital context during the COVID-19 pandemic exhibited moderate levels of workplace resilience. The low/moderate capacity for adapting to adverse situations was related to the challenges faced in the face of the unknown, the fear of exposure to the risk of COVID-19 contamination, and the impact on mental health, as evidenced by the occurrence of MPD.
