# Supplementary material for: Workplace resilience of nursing professionals during the COVID-19 pandemic: a mixed methods study
Source: Rev Bras Enferm. 2025 Sep 8;78(3):e20240155. doi: 10.1590/0034-7167-2024-0155 (PMC12419779; doi:10.1590/0034-7167-2024-0155)
Supplement: Translation

# Translation: Resiliência no trabalho de profissionais de enfermagem durante a COVID-19: pesquisa de métodos mistos

**DOI:** 10.1590/0034-7167-2024-0155pt

**Authors:** Juliana Dal Ongaro, Patrícia Bitencourt Toscani Greco, Emanuelli Mancio Ferreira da Luz, Juliana Petri Tavares, Daiane Dal Pai, Tânia Solange Bosi de Souza Magnago

**Affiliations:** I Universidade Federal de Santa Maria. Santa Maria, Rio Grande do Sul, Brasil; II Universidade Federal de Rio Grande. Rio Grande, Rio Grande do Sul, Brasil; III Universidade Federal do Rio Grande do Sul. Porto Alegre, Rio Grande do Sul, Brasil

**Keywords:** Resiliência Psicológica, Saúde do Trabalhador, Profissionais de Enfermagem, COVID-19, Pandemias

## Abstract

**Objetivo:**

Analisar a resiliência no trabalho de profissionais de enfermagem que atuaram em hospitais do sul do Brasil durante a pandemia da COVID-19 e os fatores associados.

**Métodos:**

Pesquisa de métodos mistos com design sequencial explanatório, realizada em quatro hospitais do Rio Grande do Sul. Utilizou-se um estudo transversal com 845 trabalhadores de enfermagem, seguido de um estudo exploratório descritivo com 17 desses trabalhadores. Para a etapa quantitativa, utilizou-se estatística descritiva e inferencial; para a etapa qualitativa, foi realizada análise de conteúdo temático. Os dados foram combinados por conexão.

**Resultados:**

Na análise integradora, elaboraram-se três eixos temáticos: Trabalhadores de enfermagem e os reflexos da pandemia de COVID-19; A resiliência nos diferentes cenários laborais; e Medo e impactos na saúde mental dos profissionais de enfermagem durante a atuação na pandemia de COVID-19.

**Conclusões:**

A baixa ou moderada resiliência está relacionada aos desafios do desconhecido, ao medo de exposição, ao risco de contaminação pela COVID-19 e ao impacto na saúde mental.

## INTRODUÇÃO

O ano de 2020 foi marcado pela pandemia da doença *Coronavirus Disease-19* (COVID-19), causada pelo SARS-CoV-2. A enfermagem foi uma das profissões com maiores registros entre os casos confirmados de COVID-19, seguida de médicos, agentes comunitários de saúde e farmacêuticos^(1)^. Estudo desenvolvido no Irã evidenciou que a maioria dos casos infectados pela COVID-19 ocorreu entre profissionais da enfermagem (51,3%)^(2)^. De acordo com dados disponibilizados pelo observatório da enfermagem, desde o início da pandemia até junho de 2023, no Brasil, foram registrados 62.029 casos de profissionais infectados com COVID-19, e destes, 872 profissionais (enfermeiros, técnicos e auxiliares de enfermagem) faleceram em decorrência da contaminação pelo vírus^(3)^.

A pandemia de COVID-19 trouxe acometimentos para a saúde física dos profissionais de saúde, evidenciados pela vulnerabilidade da prática laboral, com rotinas exaustivas, readaptações e exposição ao risco de contaminação^(4)^. Além desses, o uso prolongado e rotineiro dos Equipamentos de Proteção Individual (EPIs) ocasionou lesões por pressão^(5)^. Também foram observadas repercussões na saúde mental, ocasionando angústia, pânico e sintomas depressivos, principalmente em decorrência da exposição ao vírus e ao enfrentamento diário de situações estressantes^(6)^.

Nessa perspectiva, um estudo aponta que os profissionais de saúde que não possuíam vivências em emergências de saúde pública apresentaram pior desempenho em saúde mental, re-siliência e suporte social, e, consequentemente, tiveram maior tendência a apresentar sintomas como ansiedade e dificuldades interpessoais^(7)^. Também é importante ressaltar que outra dificuldade enfrentada pelos trabalhadores neste período foi a fadiga por compaixão, que ocorre como consequência de o profissional estar frequentemente exposto ao sofrimento dos pacientes, a condições de trabalho estressantes e à pouca utilização de medidas para promover o autocuidado. No entanto, a resiliência apresentou-se como fator atenuante dos aspectos negativos da fadiga por compaixão, tanto na satisfação dos profissionais quanto na qualidade do atendimento em saúde^(8)^.

Nesse contexto, a resiliência no trabalho representa uma possibilidade de se trabalhar em condições adversas e de auxiliar na manutenção da saúde física e mental, especialmente diante de situações inesperadas, como a da COVID-19. A resiliência no trabalho refere-se à capacidade de suportar ou superar adversidades e adaptar-se a essas situações^(9)^.

Estudo evidencia que a baixa capacidade de resiliência no trabalho está associada a sintomas como ansiedade, depressão e exaustão em profissionais de saúde^(10)^. Por outro lado, altos níveis de resiliência possibilitam sentimentos positivos no contexto laboral, como satisfação, realização profissional e enfrentamento de situações adversas^(10)^. Assim, a resiliência laboral pode ser um fator que favorece a promoção da saúde, bem como os aspectos saudáveis no trabalhador, e reduz os riscos que possam influenciar negativamente a saúde mental.

## OBJETIVO

Analisar a resiliência no trabalho de profissionais de enfermagem que atuaram em hospitais do sul do Brasil durante a pandemia da COVID-19 e os fatores associados.

## MÉTODOS

### Aspectos éticos

Os preceitos éticos foram respeitados conforme o Ofício Circular nº 2/2021 sobre pesquisas desenvolvidas em ambiente virtual e a Resolução 466/2012 do Conselho Nacional de Saúde. O projeto obteve aprovação do Comitê de Ética em Pesquisa, cujo parecer foi anexado à submissão. O Consentimento Livre e Esclarecido dos participantes foi obtido em formato eletrônico.

### Desenho, período e local do estudo

Pesquisa de métodos mistos, com design sequencial explanatório (QUAN → QUAL). Esta estratégia metodológica permite, por meio da etapa qualitativa, o aprofundamento dos dados estatisticamente significativos na etapa quantitativa Em relação à atribuição de peso, a prioridade foi conferida à pesquisa quantitativa. A combinação dos dados ocorreu por meio de conexão^(11)^.

O estudo foi desenvolvido em quatro instituições hospitalares, denominadas no estudo como HA (Hospital A..), HB, HC e HD. As quatro instituições se assemelhavam por serem hospitais públicos de referência no atendimento a pacientes com COVID-19 pelo Sistema Único de Saúde (SUS) do Estado do Rio Grande do Sul, bem como por serem instituições de ensino que trabalham na perspectiva do tripé do ensino superior (ensino, pesquisa e extensão). Além disso, quanto às características das instituições, apenas uma é administrada pela Empresa Brasileira de Serviços Hospitalares (EBSERH). A etapa QUAN foi desenvolvida de agosto a outubro de 2020, e a etapa QUAL, de novembro de 2022 a março de 2023.

Foram seguidas as diretrizes STROBE (*Strengthening the Reporting of Observational Studies in Epidemiology*), COREQ (*Consolidated Criteria for Reporting Qualitative Research*) e MMAT (*Mixed Methods Appraisal Tool*) (versão 2018).

### População, critérios de inclusão e exclusão

A população elegível foi composta por profissionais de enfermagem (enfermeiros, técnicos e auxiliares) distribuídos entre 2.278 do HC, 952 do HD, 2.962 do HA e 707 do HB. A partir do cálculo amostral, com auxílio do software *Power and Sample Size for Health Researchers,* versão 0.1.5^(12)^, a amostra mínima prevista para o projeto era de 534 participantes. Após o envio dos questionários por e-mail ou WhatsApp, a amostra final foi composta por 845 profissionais de enfermagem.

Na etapa qualitativa, a seleção dos participantes foi por conveniência. Foram convidados os participantes que disponibilizaram e-mail para contato na primeira etapa do estudo. A saturação teórica foi alcançada a partir da repetitividade dos depoimentos, totalizando 17 participantes nesta etapa. Neste estudo, a saturação teórica foi compreendida como o ponto de análise dos dados qualitativos em que o pesquisador constata, a partir da análise dos dados e da amostragem estabelecida, que não há o surgimento de novos fatos e que todas as teorias e conceitos subjacentes foram alcançados de acordo com o objeto de estudo, ou seja, os conceitos e os aspectos circundantes entre esses foram identificados, verificando-se que não há necessidade de adicionar dados para responder ao objeto de pesquisa^(13, 14)^.

Foram selecionados trabalhadores de enfermagem atuantes na assistência hospitalar durante o período da COVID-19. Para a etapa qualitativa, foram selecionados os participantes que disponibilizaram o e-mail para contato na etapa anterior. Excluíram-se trabalhadores afastados por qualquer motivo durante a coleta de dados.

### Protocolo do estudo

A primeira etapa (QUAN) ocorreu via plataforma Google Forms, contendo informações sociodemográficas, laborais, hábitos, o *Self-Report Questionnaire – 20* (SRQ-20)^(15)^ e a *Resilience at Work Scale 25-Brasil (RAW Scale* 25 – Brasil)^(16)^. Para esta etapa, foi utilizado um instrumento contendo informações referentes a dados gerais do trabalhador, como variáveis sociodemográficas (faixa etária, sexo, situação conjugal e número de filhos); laborais (cargo, instituição onde trabalha, tipo de vínculo trabalhista, outro vínculo, cargo de chefia, turno de trabalho, realocado de seu setor, atuação em unidade COVID-19 e atendimento a paciente com COVID-19); e hábitos e saúde (tabagismo, aumento no consumo de bebida alcoólica, atividade física, medicação iniciada na pandemia, necessidade de afastamento do trabalho por motivo de saúde durante a pandemia, afastamento do trabalho por diagnóstico de COVID-19, afastamento do trabalho por suspeita de COVID-19, medo sentido frente à exposição ao risco de contaminação, impacto na saúde física e impacto na saúde mental).

O SRQ-20^(15)^ avalia sintomas como humor depressivo-ansioso, sintomas somáticos, decréscimo de energia vital e pensamentos depressivos^(15)^. Trata-se de uma escala dicotômica (sim/não) composta por 20 questões, cujos escores variam de zero (nenhuma probabilidade) a 20 (extrema probabilidade). Já a *RAW Scale 25 – Brasil* ^(16)^ avalia a resiliência no trabalho por meio de 25 itens distribuídos em sete dimensões: vivendo autenticamente, encontrando sua vocação, mantendo o equilíbrio, administrando o estresse, interagindo cooperativamente, mantendo-se saudável e construindo redes. As respostas variam conforme a escala Likert de sete pontos, com opções de zero (Discordo Totalmente) a seis (Concordo Totalmente)^(16, 17)^. Os itens de número 9 e 11 são reversos nas análises^(17)^.

A segunda etapa do estudo (QUAL) foi realizada por entrevista, mediante um roteiro semiestruturado, orientado a partir das associações significativas com a resiliência no trabalho. As entrevistas foram conduzidas por um único entrevistador, de forma online, pela plataforma Google Meet, e gravadas em mídia digital, com duração média de 20 minutos, sendo posteriormente transcritas na íntegra.

### Análise dos resultados e estatística

A análise das variáveis, após conferência de erros e possíveis inconsistências, foi realizada no programa PASW Statistics® (*Predictive Analytics Software,* da SPSS Inc., Chicago, USA), versão 18.0. Variáveis sociodemográficas, laborais, hábitos e saúde foram analisadas por estatística descritiva. Para o SRQ-20, a suspeição de Distúrbios Psíquicos Menores (DPM) foi utilizada como ponto de corte ≥ 7, para ambos os sexos, feminino e masculino. Já para as análises da *RAW Scale 25-Brasil,* foram seguidas as orientações dos autores^(17)^. A variável resiliência no trabalho foi categorizada em níveis “Baixo”, “Moderado” e “Alto”.

Para a análise bivariada entre as variáveis independentes (sociodemográficas, laborais, hábitos e saúde, e DPM) e a dependente (resiliência no trabalho), utilizou-se o teste Qui-Quadrado de Pearson (variáveis categóricas) e a correlação de Spearman (variáveis quantitativas). Foram consideradas associações estatisticamente significativas quando p < 0,05.

Para a análise multivariada, utilizou-se a Regressão de Poisson, com variância ajustada e robusta, e a Razão de Prevalência (RP) e os respectivos intervalos de confiança (IC95%). A variável dependente foi dicotomizada em “Baixo/Moderado” e “Alto” nível de resiliência no trabalho. Variáveis de confusão foram incluídas no modelo quando apresentaram um p-valor ≤ 0,25, tanto com a exposição quanto com o desfecho.

Na etapa QUAL, foi utilizada a análise temática de conteúdo^(14)^. Os participantes foram identificados com a letra “E” de Enfermeiro, “TE” de Técnico de Enfermagem e “AE” de Auxiliar de Enfermagem, seguidas pela ordem numérica das entrevistas (Exemplo: E01, E02...; TE01, TE02...; e AE01, AE02...).

A mixagem dos dados foi realizada por conexão, ou seja, buscou-se, com os depoimentos das entrevistas, o aprofundamento e insights aos achados significativos da etapa quantitativa^(11)^. As metainferências resultantes da combinação (QUAN → QUAL) foram apresentadas por meio de joint-display (Quadro 1).

**Quadro 1:** Joint-display da conexão dos resultados QUAN (n=845) e qual (n=17) sobre a resiliência no trabalho, em profissionais de enfermagem hospitalar, durante a pandemia de COVID-19. Rio Grande do Sul, Brasil, 2020 – 2022

Eixo temático	Resultados QUAN	Resultados QUAL	Metainferências (QUAN → QUAL)
	Fatores associados ao baixo nível de resiliência no trabalho:	Entrevista semiestruturada	
Tema I – Trabalhadores de enfermagem e os reflexos da pandemia da COVID-19	- Suspeição para DPM (p < 0,001)	*Eu tive nesse ano era principalmente uma falta de disposição, uma irritabilidade, um cansaço crônico sabe, sem energia. Dores às vezes, eu me sinto assim, ó esgotada no sentido de não ter paciência, sabe? (E05 pag 8)* *A verdade nem eu sabia que eu tinha tanta, eu acho que é coragem, força. Nem eu não sabia. Porque no primeiro momento, pensei que eu não fosse conseguir vencer aquilo tudo. Mas depois eu percebi, nossa eu sou capaz! Me senti assim, até mesmo, eu acho que me fortaleceu. (AE01 pag6)*	Os profissionais de enfermagem com suspeição para DPM foram associados com o baixo/moderado nível de resiliência no trabalho. Essa associação foi confirmada na conexão dos dados, ou seja, os dados QUAN (SRQ20) foram reafirmados quando os profissionais de enfermagem elucidaram (qual) que a suspeição para os DMP ocorreu mediante a sintomatologia de fadiga, cansaço, irritabilidade, sensação de “esgotamento” que foram associados à moderada resiliência no trabalho. A conexão se fez visível mediante aspectos característicos dos domínios mantendo o equilíbrio e administrando o estresse. Estes estão presentes no sentimento de se sentir capaz, de ter força e de manter o foco, mesmo frente as adversidades advindas com a pandemia, o que pode justificar o moderado nível de resiliência no trabalho.
Tema II – A resiliência nos diferentes cenários laborais	- Pertencentes a instituição HA (p = 0,022) - Pertencentes a instituição HD (p = 0,024)	- Instituição HA *Bom eu trabalhava em um anexo da instituição e aí durante a pandemia disseram que precisavam de nós na emergência. E esse é o setor que eu menos gosto, não gosto de emergência. E eu disse para um chefe “aíme coloca em qualquer local, mas eu não gosto porque é bem desorganizado”. Então mudou completamente assim, porque era outra rotina. Não fiquei bem, porque não era o meu setor, e o que eu faço, mas se fosse ficar lá não é para mim não. (AE01, pag2)* *[...] a característica do trabalho está ficando mais difícil, os procedimentos mais complexos. E tem o agravante da gente trabalhar à noite, eu não tenho a mesma quantidade de energia. A gente nem sempre consegue fazer os intervalos. Quando a gente se deita para fazer o intervalo e já toca o telefone e aí acaba o intervalo. E isso faz diferença, tem muito esforço físico, assim tu caminhas e caminhas, resolve coisas, e tem o estresse, tem um paciente muito grave, tem uma pressão, tem uma equipe em cima de ti, tem as coisas que tu tens que dar conta naquele processo. E de madrugada tu já está cansada. O teu raciocínio não funciona, tu tens que usar uma carga de energia extra, e isso é desgastante. (E08; pag15)* - Instituição HD *Teve um período que fomos ajudar no pronto-socorro, foi um período de estresse porque íamos de outro setor com característica bem diferentes do habitual. O dia que eu tava de plantão tinha cinco pacientes em ventilação mecânica e não eram covid, era PS clínico. Isso me lembra que gerou bastante cansaço, estresse e desgaste para a equipe esse deslocamento. (E02, pagll)* *Não a instituição não substituiu ninguém. De vez em quando eles mandavam algum enfermeiro, só que não tinha experiência com a rotina, com o cuidado de paciente oncológico. Então, eu me senti muito sobrecarregado (E04, pag11)*	O baixo/moderado nível de resiliência no trabalho foi associado com profissionais de enfermagem que pertenciam a instituição HA e HD. Este achado acompanha os depoimentos sobre as particularidades que contemplam os respectivos domínios, vivendo autenticamente, interagindo cooperativamente e mantendo o equilíbrio. Dentre estes, é possível elucidar o deslocamento entre diferentes setores para atuação, a “quebra” momentânea do vínculo criado no setor atual e pela falta de apoio institucional mediante a não substituição necessária diante aos afastamentos o que ocasionou sobrecarga e desgastes na equipe; por questões que não vinham ao encontro de seus valores pessoais diante a prática laboral; pela falta de profissionais especializados para tais demandas laborais mediante a contratação emergencial de novos funcionários para suprir a alta demanda acarretada pela pandemia, o que por vezes não possibilitou desconsiderarem a experiência ou formação em especialidade. Os desafios impostos pelas mudanças são aspectos relevantes para o profissional desenvolver comportamentos resilientes mediante a determinadas situações no trabalho.
Tema III – Medo e os impactos na saúde mental dos profissionais de enfermagem durante a atuação na pandemia de COVID-19	- Raramente medo frente à exposição ao risco de contaminação pelo coronavírus (p = 0,032) - Intenso impacto na saúde mental (p = 0,014) - Alto impacto na saúde mental (p = 0,006)	*De um modo geral, o medo sempre existe, principalmente por a gente não saber como o nosso organismo vai reagir. Muitas pessoas a gente viu que pegavam, testavam positivo, mas não tinham nenhuma complicação maior do que um sintoma gripal e outras pessoas precisavam ficar internadas e evoluíam para óbito ou saiam com sequelas. (TE01; pag8)* *Preocupada, medo de não dar conta, e o cansaço mental é imenso também, porque a gente acaba tendo algum esquecimento de fazer alguma coisa, medo de cometer algum erro que possa ter alguma repercussão negativa. Então, tem afetado bastante o cansaço mental. (E01; pag7)* *O nosso maior problema foi psicológico mesmo, o medo do erro pelo cansaço. Não foi fácil de se adaptar [...]. (TE07 pag3)*	Sentir-se raramente com medo, frente à exposição ao risco de contaminação pelo coronavírus, esteve associado ao baixo/moderado nível de resiliência no trabalho. Essa associação foi confirmada nos depoimentos em que os profissionais relatam que o medo não estava centrado na autocontaminação, mas sim na transmissão do vírus. Demonstrando que uma vez apresentando raramente a sensação de medo o profissional de enfermagem não investia energia psíquica para o desenvolvimento de estratégias para as adaptações no trabalho e no cotidiano, inclusive para a necessidade de autocuidado pessoal/emocional. Os profissionais de enfermagem que tiveram intenso e alto impacto na saúde mental apresentaram baixo/moderado nível de resiliência no trabalho. Essa evidência foi ratificada nos depoimentos em que o “cansaço mental”, a preocupação excessiva, medo de não dar conta, ocasionou moderada adaptação destes profissionais às situações adversas que o trabalho na pandemia ocasionou nos serviços de saúde. Estes achados também convergem ao domínio mantendo o equilíbrio, o qual traz a necessidade de gerenciar os aspectos negativos vivenciados no trabalho, a fim de proporcionar um pensamento resiliente para conseguirem lidar com as adversidades do momento, como pelo medo da contaminação pela COVID-19, resultando, assim, um alto impacto na saúde mental.

## RESULTADOS

A etapa QUAN evidenciou que, dos 845 participantes, predominaram técnicos ou auxiliares de enfermagem (55,6%), seguidos de enfermeiros (44,4%). Destes, pertenciam à instituição HA (18,3%), à HB (10,7%), ao HC (43,4%) e ao HD (27,6%). A maior parcela era do sexo feminino (84,9%), com faixa etária entre 20 e 41 anos (50,2%), casados e/ou com companheiro (74%) e com filhos (69,0%).

Os profissionais de enfermagem foram classificados com baixo (14%), moderado (55,1%) e alto nível de resiliência no trabalho (30,9%). Os participantes apresentaram níveis moderados de resiliência no trabalho, no que tange aos domínios da *RAW Scale 25 – Brasil,* sendo: 52,1% para vivendo autenticamente, 56,3% para encontrando sua vocação, 56,0% para mantendo o equilíbrio, 47,7% para administrando o estresse, 58,5% para interagindo cooperativamente, 44,0% para manter-se saudável e 55,5% para o domínio construindo redes.

Na análise bivariada, a faixa etária de 42 a 66 anos (55,2%; p = 0,023) e profissionais que trabalham na instituição HD (60,5%; p = 0,001) apresentaram diferenças estatisticamente significativas para nível moderado de resiliência no trabalho. Ainda, não praticar atividade física (56,9%; p < 0,000), aumento no consumo de bebidas alcoólicas (55,5%; p = 0,002), não fazer uso de novas medicações na pandemia (55,5%; p < 0,000), sentir medo frequentemente diante da exposição ao risco de contaminação (62,2%; p < 0,000), alto impacto na saúde física (60,0%; p < 0,000) e mental (62,1%; p < 0,000) e péssima qualidade de sono (58,7%; p < 0,001) apresentaram associações significativas para nível moderado de resiliência no trabalho. As associações ajustadas e brutas entre a resiliência no trabalho, DPM e potenciais fatores de confusão são apresentadas na Tabela 1.

**Tabela 1:** Análises bruta e ajustadas das associações estatisticamente significativas entre a resiliência no trabalho, distúrbios psíquicos menores e potenciais fatores de confundimento, Rio Grande do Sul, Brasil, 2020

Variáveis	RPb	IC (95%)	RPajl*	IC (95%)	RPaj2**	IC (95%)	RPaj3***	IC (95%)
DPM								
Sim	1,20	1,16-1,24	1,18	1,13-1,23	1,16	1,12-1,22	1,17	1,13-1,22
Não	1	-	1	-	1	-	1	-
Afastamento do trabalho durante a pandemia								
Sim	1,02	0,98-1,06	0,99	0,96-1,03	-	-	-	-
Não	1	-	1	-	-	-	-	-
Afastamento do trabalho por COVID-19								
Não	1,06	0,99-1,13	1,05	0,99-1,12	1,05	0,99-1,11	-	-
Sim	1	-	1	-	1	-	-	-
Sexo								
Feminino	1,05	0,99-1,11	1,06	0,99-1,12	-	-	-	-
Masculino	1	-	1	-	-	-	-	-
Faixa etária								
20 a 37	1,43	0,99-1,09	1,05	0,99-1,09	-	-	-	-
38 a 44	1,02	0,98-1,07	1,03	0,97-1,08	-	-	-	-
45 a 66	1	-	1	-	-	-	-	-
Instituição onde trabalha								
HA	1,07	1,02-1,12	1,06	1,01-1,11	1,06	1,01-1,06	1,06	1,00-1,11
HB	1,04	0,98-1,11	1,04	0,97-1,10	1,03	0,97-1,09	1,03	0,98-1,09
HD	1,02	0,98-1,07	1,03	0,97-1,08	1,06	1,01-1,10	1,01	1,00-1,09
HC	1	-	1	-	1	-	1	-
Turno de trabalho								
Sem turno fixo	1,02	0,94-1,11	1,04	0,96-1,13	1,00	0,92-1,09	-	-
Noturno	0,96	0,92-1,00	0,97	0,93-1,01	0,99	0,95-1,03	-	-
Diurno	1	-	1	-	1	-	-	-
Medo sentido frente à exposição ao risco de contaminação								
Frequentemente	1,24	1,09-1,40	1,25	1,10-1,41	1,08	0,96-1,22	1,11	0,99-1,26
Raramente	1,18	1,03-1,34	1,18	1,03-1,34	1,11	0,98-1,26	1,15	1,01-1,30
Ocasionalmente	1,16	1,02-1,31	1,16	1,03-1,31	1,07	0,95-1,21	1,09	0,97-1,24
Muito	1,23	1,09-1,39	1,24	1,09-1,39	1,06	0,93-1,19	1,08	0,95-1,22
Nenhum	1	-	1	-	1	-	1	-
Atividade física								
Não	1,08	1,04-1,13	1,06	1,02-1,10	1,03	0,99-1,07	-	-
Sim	1	-	1	-	1	-	-	-
Aumento do consumo de álcool na pandemia								
Sim	1,05	1,01-1,09	1,05	1,01-1,09	1,03	0,99-1,07	-	-
Não	1	-	1	-	1	-	-	-
Medicação iniciada na pandemia								
Sim	1,06	1,01-1,09	0,99	0,96-1,04	-	-	-	-
Não	1	-	1	-	-	-	-	-
Impacto na saúde física								
Intenso	1,17	1,07-1,30	1,14	0,94-1,15	1,04	0,95-1,15	-	-
Pouco	1,13	1,01-1,27	1,02	0,99-1,23	1,09	0,98-1,21	-	-
Moderado	1,15	1,04-1,27	1,08	0,98-1,19	1,08	0,98-1,19	-	-
Alto	1,16	1,05-1,29	1,01	0,91-1,12	1,00	0,90-1,12	-	-
Nenhum	1	-	1	-	1	-	-	-
Impacto na saúde mental								
Intenso	1,23	1,12-1,35	1,14	1,03-1,26	1,15	1,04-1,28	1.13	1,03-1,25
Nenhum	0,98	0,84-1,14	1,01	0,87-1,18	1,05	0,89-1,24	1,00	0,86-1,18
Moderado	1,11	1,00-1,22	1,09	0,99-1,19	1,09	0,99-1,21	1,08	0,98-1,19
Alto	1,20	1,09-1,32	1,14	1,04-1,26	1,14	1,03-1,26	1,14	1,04-1,25
Pouco	1	-	1	-	1	-	1	-
Qualidade do sono								
Péssima	1,26	1,14-1,39	1,22	1,10-1,34	1,09	0,99-1,21	-	-
Ruim	1,19	1,08-1,30	1,15	1,05-1,27	1,05	0,96-1,15	-	-
Regular	1,15	1,05-1,26	1,23	1,03-1,23	1,03	0,95-1,31	-	-
Bom	1,11	1,01-1,22	1,09	0,99-1,19	1,05	0,95-1,15	-	-
Ótima	1	-	1	-	1	-	-	-

*Legenda: DPM: Distúrbios Psíquicos Menores; RPb: Razão de Prevalência bruta. RPaj: Razão de Prevalência ajustada. IC (95%): Intervalo de Confiança.*

Nota: RP_aj1*_= DPM + afastamento do trabalho durante a pandemia + afastamento do trabalho por COVID-19 + faixa etária + instituição onde trabalha + turno de trabalho + realocado de setor + atividade física + aumento do consumo de álcool + medicação iniciada na pandemia + atender paciente COVID-19 + Medo sentido frente à exposição ao risco de contaminação + impacto na saúde física + impacto na saúde mental + qualidade do sono; RP_aj2**_ = Afastamento + DPM + Resiliência no trabalho + instituição onde trabalha + turno de trabalho + medicação iniciada na pandemia + impacto na saúde física + impacto na saúde mental; RP_aj3***_= DPM + instituição onde trabalha + medicação iniciada na pandemia + impacto na saúde física + impacto na saúde mental.

Quanto aos DPM, profissionais de enfermagem com suspeição, durante a pandemia pela COVID-19, apresentaram prevalência mais elevada de 17% para baixa resiliência no trabalho. Ainda, profissionais pertencentes às instituições HA e HD apresentaram, respectivamente, prevalências de 6% e 1% mais elevadas para baixa resiliência no trabalho, quando comparadas à instituição HC.

Os profissionais de enfermagem que referiram raramente sentir medo diante da exposição ao risco de contaminação pela COVID-19 apresentaram prevalência de 15% mais elevada para baixa resiliência no trabalho. Profissionais que relataram um intenso e alto impacto na saúde mental apresentaram prevalências de 13% e 14%, respectivamente, mais elevadas para baixa resiliência no trabalho.

Na etapa qualitativa, predominou a participação do sexo feminino (82,3%), na faixa etária de 40 a 50 anos (47,0%) e com tempo de trabalho de 5 a 7 anos (64,7%). Destes, oito eram enfermeiros, oito técnicos e um auxiliar de enfermagem. Ainda, três profissionais eram provenientes da instituição HA, dois da HB, seis da HC e seis da HD. Além disso, oito profissionais de enfermagem relataram que não precisaram ser realocados de setor/unidade de atuação, quatro foram realocados quando necessário e cinco foram realocados de setor/unidade de atuação para atender especificamente às demandas da pandemia pela COVID-19.

Os dados qualitativos possibilitaram a construção de duas categorias temáticas: “Reflexos do enfrentamento da pandemia de COVID-19 na saúde dos profissionais de enfermagem” e “Capacidade de adaptação e estratégias para manter a saúde durante a atuação na pandemia de COVID-19”. A primeira apontou reflexos de uma nova rotina, com necessidade de remanejamento para a atuação em novos setores de trabalho, com recursos humanos insuficientes, sobrecarga horária e escassez dos EPI’s, principalmente no início da pandemia. Também evidenciou-se repercussões advindas do estresse, vivenciado pelo medo e pelo esgotamento diante do receio de transmissão viral para algum familiar.

*Muita sobrecarga para todos. Foi uma coisa incontrolável porque a escala hoje era com 20 funcionários, aí quando chegava para trabalhar, tinha menos cinco. Não tinha como substituir aqueles cinco naquele momento. E aí, se trabalhava com que tinha. A instituição começou a fechar unidades por não ter mão de obra para ajudar e a substituição não era a mão de obra que tivesse o conhecimento, era ajuda ali.* (TE08; pag4)

*Me sinto fadigada, desmotivada* [...] *não é sempre assim a ponto de uma depressão, eu adoro meu trabalho, gosto de ir, mas por vezes me sinto cansada* (E02; pag10)

Na segunda categoria, foi possível elaborar duas subcategorias. Na primeira “O papel da resiliência para manutenção do bem-estar laboral e pessoal”, emergiram aspectos sobre a importância da resiliência para a manutenção do bem-estar laboral e pessoal, conforme depoimentos:

*Acho que a gente, um ao outro, vamos nos fortalecendo, porque nós vamos nos cuidar e a gente tem que estar bem para seguir adiante. Então, eu acho que a gente se fortalece e se fortaleceu mais ainda enquanto equipe, um apoiando o outro, e as que tiveram a covid a gente se preocupava, ligava e procura acompanhar, mesmo a distância.* (E03; pag5)

[...] *a gente tem momentos que se sente com o emocional abalado, mas a gente tem uma resiliência incrível. Tem uma força maior, que te permite lutar e enfrentar novamente. A gente renasce todos os dias. Eu acho que é a resiliência mesmo. É você ter que achar forças, é você ter que ser forte diante de momentos difíceis.* (TE08; pag12)

A segunda subcategoria “Estratégias para a promoção à saúde num contexto de pandemia” apontou que a prática de leitura, a busca por estudar conteúdos novos, o artesanato, a meditação, a prática de atividade física em casa constituíram estratégias para “manter o corpo ativo”. Estas atividades auxiliaram a manter o foco, a concentração e a manutenção de uma rotina saudável, tanto do ponto de vista físico quanto mental.

*Eu comecei a praticar meditação e notei que me ajudou a focar mais no trabalho e que aquele momento que estávamos passando não interferisse na minha saúde.* [...] *Então, eu consegui trabalhar mais com o corpo e eu usei a mente junto.* (AE01; pag4)

[...] *procurava ler e fazer as coisas: exercício físico, relaxamento em casa, mas a oração para manter a fé foi um ponto forte em mim.* (TE07; pag7)

No Quadro 1, apresenta-se o *joint-display* com a combinação dos resultados por conexão (QUAN → qual). A partir disso, obtiveram-se meta-inferências e *insights* únicos sobre a resiliência no trabalho e as associações significativas entre fatores de exposição em profissionais de enfermagem hospitalar, durante a pandemia da COVID-19.

## DISCUSSÃO

A mixagem dos dados foi concordante no que tange à suspeição para os DPM, em que profissionais de enfermagem apresentaram baixo/moderado nível de resiliência no trabalho (p < 0,001). Os participantes referiram fadiga, irritabilidade, falta de concentração e energia, cansaço físico e psíquico. Esses são sinais e sintomas condizentes para suspeição de DPM^(15)^.

Os sintomas de DPM também estiveram presentes no que concerne à exposição e ao risco de contaminação, além da vulnerabilidade de adoecimento grave entre os profissionais de enfermagem investigados. Estudos brasileiros, com a mesma população e cenário, também evidenciaram sinais e sintomas preocupantes para os DPM, com prevalências de 35,5%^(18)^ a 60,6%^(19)^. Cabe considerar que é provável que o contexto pandêmico tenha contribuído para o agravamento do adoecimento laboral entre os profissionais de enfermagem, uma vez que, mesmo antes da pandemia, já se destacava um cenário desfavorável na saúde desses profissionais, com reflexos tanto nos aspectos da saúde física quanto mental.

Estudo iraniano evidenciou que profissionais que não possuem a capacidade de resiliência no trabalho tendem a apresentar seis vezes mais chances de desenvolver algum tipo de distúrbio psicológico (p = 0,02)^(20)^. Como exemplo, destacam-se a ansiedade^(21)^, o desgaste emocional e a despersonalização^(22)^. Conseguir desenvolver essa capacidade de adaptação em situação adversa, como a vivenciada na pandemia da COVID-19, permite ao profissional de enfermagem minimizar os efeitos negativos, proporcionando qualidade do sono e maior satisfação laboral^(23, 24)^.

Outrossim, a resiliência é um fator crucial para conseguir se manter e se adaptar diante dos novos desafios e adversidades, podendo ter reflexos tanto na vida pessoal quanto na laboral. Para este processo adaptativo, há a necessidade de administrar o estresse, de reconhecer seus pontos fortes e de conseguir colocá-los em prática no trabalho, de redes de apoio, seja no trabalho ou na esfera pessoal, e de buscar momentos que possam favorecer a saúde física e mental. Assim, a avaliação da resiliência constitui uma importante estratégia para o autoconhecimento dos trabalhadores, possibilitando que a instituição possa planejar ações de promoção da resiliência, de saúde e de qualidade de vida^(16)^.

Neste ínterim, na etapa integradora, inicialmente foi notado que aspectos dos domínios “Mantendo o equilíbrio” e “Administrando o estresse” corroboraram com o baixo/moderado nível de resiliência no trabalho apresentado pelos profissionais investigados. Estes domínios são reflexos de características como conseguir manter o foco, gerenciar as situações desfavoráveis, manter rotinas e equilíbrio entre a vida profissional e a pessoal^(25)^, mesmo diante de situações adversas, como na pandemia. Enfatiza-se o cenário totalmente desconhecido neste período, o qual exigiu dos profissionais de enfermagem adaptação a novos processos de trabalho, com a intensificação das atividades assistenciais e com alta demanda psíquica, na qual o cansaço físico e mental foram visíveis, o que pode estar atrelado ao nível de resiliência apresentado por esses profissionais.

Quanto à evidência de que o baixo/moderado nível de resiliência no trabalho esteve associado a profissionais pertencentes às instituições HA e HD, destaca-se que ambas possuem como particularidade o atendimento 100% ao SUS. Nesse cenário, diversas mudanças foram inevitáveis para que as instituições de saúde pública enfrentassem a crise sanitária e econômica vivenciada.

Os achados da fase qualitativa apoiaram os achados quantitativos e também forneceram significados contextuais laborais, ou seja, foi possível a interlocução com aspectos referentes à resiliência. Em especial, aspectos relativos à possibilidade de conhecer e manter valores e pontos fortes pessoais, um bom nível de consciência emocional e regulação no espaço de trabalho; com a obtenção de apoio mútuo entre colegas; e a capacidade de vencer contratempos, melhorar habilidades, atualizar-se em conhecimentos especializados e expandir as redes convergem com os domínios vivendo autenticamente, mantendo o equilíbrio e interagindo cooperativamente^(25)^.

Ficou evidente a dificuldade enfrentada pelos profissionais para a troca de setor de trabalho, principalmente quando não há uma identificação com o mesmo, ou ainda, quando não há conhecimento específico para tal atuação, o que pode acarretar insegurança, sobrecarga e desgaste no trabalhador. Além disso, os remanejamentos dos profissionais que compunham os grupos de risco acarretaram contratações emergenciais e a dissolução de equipes, o que, por sua vez, exigiu a construção de novos vínculos e redes de apoio, com impacto no desenvolvimento de comportamentos resilientes.

Estudo destaca que o rompimento de vínculos afetivos existentes nas equipes de trabalho, em detrimento de novos dimensionamentos e redistribuições dos profissionais, configurou-se como um momento sensível, com implicações no apoio social que já existia, exigindo a construção de novos vínculos e de uma organização do trabalho com colegas novos^(26)^.

Além disso, tempo de experiência (p = 0,01) e área de atuação possuem relação significativa e positiva para a capacidade de resiliência no trabalho da enfermagem^(27)^. O despreparo profissional diante das situações adversas favorece o surgimento de condições estressantes e, com isso, os profissionais não conseguem progredir e aperfeiçoar suas habilidades, o que poderia facilitar sua adaptação de forma mais resiliente. Além disso, as substituições de colegas para atender demandas que não são de sua rotina podem implicar no risco de contaminação e transmissão, representando um fator estressor entre os trabalhadores^(28)^ e, assim, comprometendo a qualidade do atendimento prestado à população^(29)^. Cabe elucidar que a capacidade de resiliência no trabalho pode possibilitar ao profissional desenvolver uma assistência mais segura diante de um cenário desconhecido, como o presenciado pela pandemia da COVID-19^(30)^.

Ainda, os resultados evidenciaram que profissionais de enfermagem que referiram raramente sentir medo da exposição ao risco de contaminação pelo coronavírus (p = 0,032), bem como intenso (p = 0,014) e alto (p = 0,006) impacto na saúde mental, apresentaram prevalências para baixa/moderada capacidade de resiliência no trabalho. Esses dados se confirmam diante dos depoimentos, nos quais o medo não estava centrado na exposição diária, mas em momentos de incerteza diante da assistência, por não saberem o diagnóstico prévio dos pacientes assistidos, ocasionando receio de transmissão do vírus aos familiares. Esse achado elucida que a sensação de medo ao atuar diante da COVID-19 talvez não tenha proporcionado, aos profissionais de enfermagem, um impulso protetivo, considerando as estratégias de adaptações voltadas para o trabalho. Também observou-se o cansaço mental como um empecilho para a adaptação às situações adversas no trabalho desenvolvido na pandemia.

Em consonância com as evidências elencadas, um estudo evidenciou que o medo de transmitir a doença para familiares ou amigos, a incerteza percebida sobre o tratamento, a indisponibilidade de recursos, como testes rápidos e EPIs, constituíram fatores desencadeadores de angústia entre os profissionais de enfermagem, majoritariamente acometidos^(31)^. Além disso, um estudo multicêntrico com enfermeiros atuantes em hospitais de referência para o atendimento à COVID-19 identificou que o medo frente à exposição ao risco de contaminação esteve associado ao desenvolvimento de DMP (p < 0,001)^(19)^. Ou seja, aspectos como o medo tornam-se um potencializador para gerar impactos negativos na saúde mental desses profissionais^(32)^, incapacitan-do-os de traçar adaptações positivas em suas jornadas laborais.

Ainda, reflexos na saúde mental foram evidenciados diante do surgimento da síndrome de burnout (p = 0,007), sintomas graves de ansiedade (RP = 1,36; p = 0,019) e depressão (RP = 1,40, p = 0,011) em profissionais de enfermagem com atuação direta na linha de frente de enfrentamento à pandemia^(33)^.

Os achados deste estudo vêm ao encontro de características que tangenciam o domínio “mantendo o equilíbrio” da *RAW Scale.* Este descreve a necessidade do profissional em gerenciar a negatividade vivenciada no trabalho e, assim, potencializar a capacidade de resiliência para lidar com as adversidades do momento^(25)^. Ou seja, o medo da exposição à contaminação, sentido pela atuação na pandemia da COVID-19, resultou em impacto na saúde mental dos profissionais de enfermagem, os quais, de alguma forma, não conseguiram desenvolver adaptações, sejam elas pessoais ou laborais, que pudessem favorecer o enfrentamento das adversidades do momento.

Estudo com profissionais de enfermagem, com assistência direta a pacientes durante a COVID-19, de todas as regiões do Brasil, evidenciou que baixos níveis de resiliência impactaram nas maiores pontuações médias para depressão^(34)^. Em suma, demonstra a resiliência como um fator essencial para a proteção à saúde laboral, reduzindo impactos como desgaste emocional, exaustão física, sobrecarga, medos, preocupações e incertezas advindas de um contexto como o da COVID-19. Em consonância, profissionais de saúde com altos índices de resiliência no trabalho conseguiram se adaptar às situações adversas impostas pela vasta pandemia da COVID-19^(10)^.

Estudo chinês avaliou métodos de melhorias para reduzir o impacto dos problemas psicológicos decorrentes da atuação na pandemia de COVID-19 pela equipe de enfermagem. Estes contemplaram a realização de medidas de avaliações e de intervenções precoces para aliviar a ansiedade, o estresse e a tensão; o incentivo à criação de vínculos e ao desenvolvimento de um trabalho em harmonia para evitar o estresse; e a melhoria no apoio social recebido^(35)^. Nesta perspectiva, emerge a importância da promoção de aspectos positivos no trabalho. Dentre estes, destaca-se a capacidade de resiliência, tendo em vista as repercussões negativas na saúde psíquica advindas dos ambientes laborais, especialmente no recorte da pandemia. Essas repercussões podem ocasionar prejuízos tanto na qualidade de vida individual quanto na assistência prestada aos indivíduos, à família e à comunidade.

### Limitações do estudo

Como limitação, destaca-se o intervalo de tempo entre as etapas quantitativa e qualitativa do estudo. As entrevistas foram realizadas inteiramente de forma online, porém as demandas por interações remotas passaram a sobrecarregar os profissionais, os quais muitas vezes não se dispunham a participar ou a permanecer em frente ao computador ou a outro meio digital. Assim, o autorrelato pode ter sofrido o impacto da virtualidade. No entanto, por terem sido utilizados os métodos quantitativo e qualitativo, as fragilidades inerentes a ambos foram minimizadas, visto que os pontos positivos de uma abordagem compensaram os da outra^(11)^.

### Contribuições para a área da enfermagem, saúde ou política pública

As evidências desta pesquisa possuem importantes contribuições no campo da enfermagem e da saúde, tendo em vista o diagnóstico situacional de uma realidade laboral inesperada pela enfermagem e pela sociedade. Também, o estudo elenca a capacidade de resiliência no trabalho como sendo um fator essencial para o enfrentamento das adversidades no trabalho da enfermagem. Torna-se importante refletir sobre estratégias que contribuam para o campo da saúde e da enfermagem, que possibilitem a parceria entre serviços de saúde e a formulação de políticas públicas, com ênfase no cuidado integral desses profissionais. Nos ambientes laborais, devem ser pensadas estratégias para a promoção da resiliência e bem-estar baseadas em evidências, como o uso do *mindfulness* e a terapia cognitivo-comportamental^(36)^, as quais têm se mostrado aspectos importantes para ampliar os níveis de resiliência^(37)^, em prol da saúde dos trabalhadores. Também, é indispensável que a gestão das instituições busque medidas que promovam ambientes de trabalho saudáveis e que favoreçam o desempenho laboral mais positivo.

## CONCLUSÕES

Os profissionais de enfermagem atuantes no contexto hospitalar da pandemia da COVID-19 apresentaram moderado nível de resiliência no trabalho. A baixa/moderada capacidade de adaptação às situações adversas esteve relacionada aos desafios vivenciados frente ao desconhecido, ao medo de exposição ao risco de contaminação pela COVID-19 e ao impacto na saúde mental, representado pela ocorrência de DPM.
